# Optimizing family building in women of advanced reproductive age: a Faster-Fewer-and-Finished approach

**DOI:** 10.3389/fendo.2026.1845946

**Published:** 2026-09-04

**Authors:** Sesh K. Sunkara, Christos Venetis, Thomas D’Hooghe, Francesca E. Duncan, Tamara Hunter, Susana Montenegro, Frank Broekmans

**Affiliations:** 1 Division of Women’s Health, Faculty of Life Sciences and Medicine, King’s College London, London, United Kingdom; 2 King’s Fertility London, London, King’s College London, London, United Kingdom; 3 Unit for Human Reproduction, 1st Department of OB/Gyn, Medical School, Faculty of Health Sciences, Aristotle University of Thessaloniki, Thessaloniki, Greece; 4 Centre for Big Data Research in Health, Faculty of Medicine & Health, University of New South Wales, Sydney, NSW, Australia; 5 Merck Healthcare KGaA, Darmstadt, Germany; 6 Department of Development and Regeneration, Laboratory of Endometrium, Endometriosis & Reproductive Medicine, KU Leuven, Leuven, Belgium; 7 Department of Obstetrics, Gynecology, and Reproductive Sciences, Yale University Medical School, New Haven, CT, United States; 8 Department of Obstetrics and Gynecology, Feinberg School of Medicine, Northwestern University, Chicago, IL, United States; 9 Monash IVF, Perth, WA, Australia; 10 WOOM Women’s Health, Perth, WA, Australia; 11 Professor Em. in Reproductive Endocrinology and Surgery, Department of Reproductive Medicine, at the University Medical Center Utrecht, Utrecht, Netherlands; 12 Consultant Reproductive Medicine, Center for Reproductive Medicine, Dijklander Hospital, Purmerend, Netherlands

**Keywords:** advanced reproductive age, expert opinion, medically assisted reproductive technology, time to live birth, treatment strategy, family building, 3F approach

## Abstract

Delayed parenthood is a defining feature of contemporary reproductive medicine, fundamentally reshaping reproductive planning and increasing reliance on medically assisted reproduction (MAR). For women of advanced reproductive age (ARA), this shift exposes a potential disconnect between conventional treatment pathways and the biological constraints of ovarian ageing and a rapidly narrowing reproductive window. This risks preventable delays, cumulative treatment burden, and suboptimal outcomes in this population. Addressing these challenges requires deliberate adaptation of current clinical strategies to minimize avoidable delays in assessment and treatment, while ensuring timely, optimized, and outcome-focused care. In this Perspectives article, which is based on expert consensus opinion, we propose a hypothesis-generating framework — the Faster-Fewer-and-Finished (3F) approach — in which expedited evaluation, tailored cycle sequencing, and a patient-tailored ‘finish’ criterion are applied to maximize time-to-first-live birth or to arrive at a shared decision on how or if to continue, with treatment decisions anchored by individual age and ovarian reserve. The 3F approach prioritizes achieving a live birth or ideal family size in the shortest possible time by advocating the ‘fewest’ number of ovarian stimulation cycles necessary. While more than one cycle may be unavoidable, the emphasis is on early strategic tailoring of treatment according to age category, efficiency of intervention, and minimizing biological and emotional attrition. By aligning treatment strategies with both the realities of ovarian ageing and the wishes and life priorities of ARA patients, the 3F approach integrates established principles of MAR into a time-critical, outcome-driven theoretical framework for infertility care.

## Introduction

There is a global trend towards delayed parenthood, with the average age of first-time mothers rising steadily. In 2020, women in many Asia–Pacific countries, including Australia, Singapore, South Korea, Japan and New Zealand, had their first child at an average age of 30 years or older (1). In the European Union, the average age increased from 29.0 years in 2001 to 31.1 years in 2022 (2), with Italy and Spain reporting the oldest first-time mothers (3). Similarly, in the United States the average age increased from 21.4 in 1970 to 27.1 in 2020 (4).

The global rise in delayed parenthood is driven by societal shifts, such as the increased participation of women in higher education and the workforce, greater access to contraception, evolving societal norms, and economic factors (5). As a result, a growing number of individuals are entering advanced reproductive age (ARA), typically defined for women as ≥35 years of age. This age threshold is clinically significant as time to pregnancy (TTP) is inherently age dependent, and female reproductive potential declines significantly from this age (6, 7), with notable increments in fertility decline occurring between 40 to 43 years and again at >44 years (8). The progressive decline in fertility is due to decreased ovarian reserve and, more critically, declining oocyte quality driven by increased aneuploidy rates and impaired cytoplasmic maturation (9, 10). This places ARA women at a considerably high risk of adverse reproductive outcomes, including infertility, pregnancy loss, fetal anomalies, obstetric complications, or stillbirth (11). Consequently, delaying childbearing can lead to challenges in conceiving and increased infertility rates (12). Recent data have shown that ~63% of *in-vitro* fertilization (IVF) cycles in North America (13) and ~56% of cycles in Europe (14) are undertaken in ARA women. However, this population faces suboptimal outcomes from medically assisted reproduction (MAR) interventions compared with younger women (15), as MAR success rates also decline with age (16). This contributes to the stress and financial burden for individuals looking to build a family using this option (17) and the high levels of dropout (18).

Despite this, the decision to postpone family building is frequently underinformed and the ability of ART to overcome age-related fertility decline is overestimated (19–23). Current treatment guidelines include sections on ARA women, with the European Society of Human Reproduction and Embryology (ESHRE) guideline providing detailed, evidence-graded recommendations on specific areas such as fertility preservation (24), whereas the American Society for Reproductive Medicine guideline focusses on risk-benefits and ethical considerations of late-age family building (25).

In this Perspectives article, we set out a conceptual, hypothesis-generating framework to reduce the time to live birth in ARA women—the Faster-Fewer-and-Finished (3F) approach— developed through author discussion following a symposium and based on expert opinion informed by the published literature. This refreshed paradigm of care aims to maximize pregnancy potential with each ART cycle, resulting in fewer stimulation cycles and shorter time to achieve the desired outcome of family building, and is informed by patient autonomy, emotional preparedness and individual desires with respect to ideal family size. The “Finished” component is a patient-defined endpoint that may, on one hand, reflect achievement of the patient’s desired family-building goal. On the other hand, following shared decision-making, it may also reflect the decision to stop, pause or change strategy because of prognosis, previous treatment response, medical or obstetric risk, emotional burden, financial or access constraints, changing personal priorities, or a preference for donor gametes or another family-building pathway. We envisage these combined approaches may help clinicians to better support ARA women.

## Current strategies to reduce the time to live birth for ARA women

As time is the most critical factor in ARA women, current interventions need to be re-examined to reduce the time to live birth. To achieve this, the patient journey may generally be adapted in several ways (**Supplementary Figure 1**), including advice on timely intervention, comprehensive evaluation, early diagnosis and intervention, personalized treatment plans, and by adopting a psychosocial, multidisciplinary approach. Collaborations with other specialists, such as andrologists or obstetricians, should be considered for pre-pregnancy assessment. Although recognized preventive measures, such as smoking cessation and losing weight, may still be beneficial for ARA women, this population could also benefit from clear information that helps them make rapid, well-informed decisions on when or whether to start MAR treatment. However, there are only few high-quality guidelines that address counselling on age-related fertility decline, and the existing guidance is inconsistent (23).

## One-and-done: an approach to optimize ART cycle number to maximize pregnancy potential

A retrospective, single-center, cohort modelling study including 16,474 IVF cycles showed that it was possible to achieve 1 or ≥2 live births from a single retrieval cycle followed by use of all embryos in subsequent frozen cycles (the One-and-Done approach). With current MAR practices, over one-quarter of women could complete their family by achieving at least two live births from a single ovarian stimulation (OS), assuming that all retrieved embryos have equal potential to achieve a live birth (26).

The success of the One-and-Done approach is influenced by the patient’s age, ovarian reserve status, and oocyte quality. As a higher number of retrieved oocytes is associated with a higher number of high-quality embryos (27), younger patients with good ovarian reserve are more likely to benefit from this strategy, as they are likely to produce more euploid embryos from a given oocyte yield compared with ARA women (28). Thus, its effectiveness may diminish with advancing reproductive age. The One-and-Done approach may, therefore, be a more realistic option for women aged <35 years, in whom the ideal 15–20 oocytes (29) can be retrieved from a single fresh OS cycle. Nevertheless, data suggest that with 10–14 oocytes retrieved, around 10% of women between 38–40 years of age could still achieve at least two live births (26).

## Not all ARA women are the same

Although female fertility starts to decline significantly after the age of 35 years, further increments in fertility decline can occur between 40 to 43 years and again at >44 years (8). Accordingly, conventional treatment approaches in ARA women risks preventable delays, cumulative treatment burden, and may result in failure in women for whom time is the most critical determinant of success. We propose that treatment recommendations should, therefore, be based on a more nuanced categorization across different age ranges while also considering the desired ideal family size.

**Figure 1:**
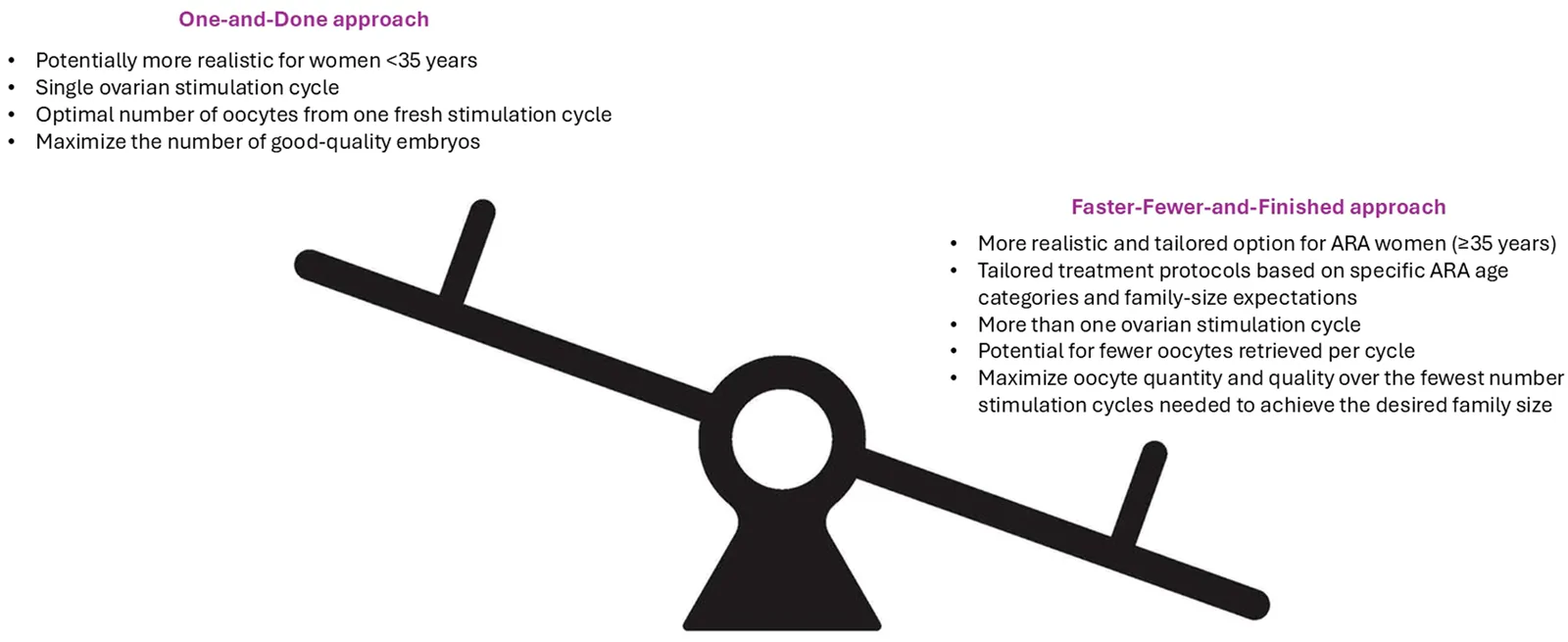
Key features of the One-and-Done and Faster-Fewer-and-Finished approaches.

In our opinion, the key to addressing this requires the minimization of avoidable delays in current strategies for assessment and treatment and minimizing delays between cycles, while still ensuring timely, optimized, and outcome-focused care. A personalized treatment plan based on an individual’s circumstances and preferences can then be adopted. Such a plan should be fully informed by the prospect that any delay in treatment may further reduce the chances of having a first baby with MAR, and potentially having a second baby in the future (30). In light of this, we propose the hypothesis-generating Faster-Fewer-and-Finished (3F) approach as an extension of the One-and-Done approach that is a more tailored and realistic option for ARA women (**Figure 1**).

## Faster-Fewer-and-Finished (3F): a refreshed paradigm of care for ARA women

The 3F concept is designed to minimize the number of stimulation cycles needed to achieve the optimal number of oocytes, giving the best chance of achieving their ideal family size. This can be accomplished by following safe and efficient protocols that optimize both oocyte quantity and quality over the shortest possible timeframe, aligned according to age category and family expectations (**Supplementary Figure 2**).

### Family expectations

A key consideration is to understand the ideal family size of each woman (i.e., do they see their family as being complete with one child or more than one child) (**Table 1**). Meeting these expectations will require further tailoring of the treatment protocols. Although elective family preservation and third-party reproduction are adjacent and important options, our focus in this Perspectives article is on women presenting for MAR with the immediate goal of building their family.

**Table 1:** The 3F treatment strategy encompassing ARA age categories and desired family size.

Age categories	35-39 years		40-43 years		44-50 years	
Family expectations	1 child	>1 child	1 child	>1 child	1 child	>1 child
Pretreatment evaluation timescale	Expedite access to treatment, to reduce the number of cycles and mitigate the decline in oocyte quality	Prioritize access to treatment, to ensure a sufficient number of back-to-back cycles can be completed to bank oocytes, to mitigate the decline in oocyte quality	Urgent access to treatment, to ensure one cycle, or more, can be completed in the shortest time	Very urgent evaluation and work-up minimise delays between back-to-back cycles, to reduce the number of cycles needed to accumulate sufficient euploid blastocysts	Consider a donor oocyte cycle (compliant with local regulations)	Consider expedited sequential donor oocyte cycles (compliant with local regulations)
Estimated number of oocytes to obtain	≥1 euploid blastocyst^*^: 11-24		≥1 euploid blastocyst^*^: 29-52		Not applicable	
	≥3 euploid blastocysts^†^: >50		≥3 euploid blastocysts^†^: >50		Not applicable	
Treatment	Gonadotropin type^‡^: r-hFSH or rhFSH + r-hLH		Gonadotropin type^‡^: r-hFSH or rhFSH + r-hLH		Not applicable	
	Starting dose^¶^: 225–300 IU/day FSH dose (although this should be based on predicted patient response and clinical judgement); within cycle dose adjustment can be considered, depending on response (up to a maximum dose of 450 IU/day)^#^		Starting dose^¶^: 225-300 IU/day FSH dose (although this should be based on predicted patient response and clinical judgement); within cycle dose adjustment can be considered, depending on response (up to a maximum dose of 450 IU/day)^#^		Not applicable	
	Duostim^**^: Could be considered if indicated (to accumulate oocytes or embryos when fresh transfer is not planned)		Duostim^**^: May be considered to accelerate egg retrieval and potentially reduce the number of full cycles needed; however, delays due to the loss of potential for a fresh transfer should be considered		Not applicable	
Finish^¶¶^	Consider this course until the desired outcome is achieved or the next milestone age is reached		Consider this until the desired outcome is achieved or consider an upper age limit in light of the increasing individual maternal health and potential obstetric risk potential		Consider this course until the desired outcome is achieved or the next milestone age is reached	
Excess embryo policy	Retain in case the expectations of the couple change or proceed in accordance with local regulations. Dispositional discussions should be part of the pre-treatment counselling process					

Recommendations based on one pregnancy; the number of back-to-back cycles required to bank the requisite number of euploid blastocysts will depend on the completed family size.

*Number of MII oocytes needed at age of vitrification giving ≥90% probability of ≥1 euploid blastocysts calculated using the Merck Oocyte Number Estimator ONE using retrospective cohort data (31, 32).

†Estimates of minimum number of MII oocytes needed by age at vitrification giving ≥99% probability of ≥3 euploid blastocysts based on population-level counselling estimates derived from a published prediction model based on retrospective cohort data (33). Oocyte yield and modelled euploid-blastocyst yield are intermediate outcomes and do not guarantee live birth or completion of the patient’s family-building goal. The actual number of oocytes and cycles required (achieved through multiple cycles and banking of oocytes) will vary according to ovarian reserve, prior ovarian response, laboratory performance, sperm-related factors and other individual characteristics. These data were obtained from cycles with PGT-A and intracytoplasmic sperm injection of MII oocytes and should be interpreted accordingly.

‡ Based on EHSRE 2025 Guidelines for OS (34) and informed by Conforti 2021 and Bielfeld 2023.

¶ Based on Lensen 2018 (84) and Ngwenya 2024 (85).

# Within cycle dose adjustments are probably not recommended, according to the latest ESHRE Guidelines (2025).

**Based on EHSRE 2025 guidelines for OS (34) and depending on patient characteristics (e.g., very low reserve, urgent need to bank oocytes), although there is no evidence that it improves live birth rates in ARA women.

¶¶ Within the 3F approach, ‘Finished’ is defined operationally as the point at which (i) the woman’s pre-specified desired family size has been achieved with a live birth or live births, or (ii) the next age threshold is reached at which the benefit–risk balance shifts materially (40 years for the 35–39 group; 44 years for the 40–43 group), at which point the strategy and prognosis should be revisited with the patient. The rationale for an age-anchored stop is the steep age-related decline in euploid blastocyst yield per stimulation cycle and the parallel rise in obstetric and perinatal risk, rather than a fixed numerical cap on cycles.

### Age categories

Women aged >35 years should not be considered as a homogenous group, and treatment recommendations should be considered according to specific age categories as well as their desired family size. We propose separate treatment protocols for women who fall within three age categories (35–39 years, 40–43 years and 44–50 years) (8). These proposed age categories are pragmatic counselling strata intended to prompt reassessment and individualization, rather than absolute treatment thresholds, and are based on fertility status, potential for obstetric complications with increasing age, and whether their family would be complete with one child or more than one child (**Table 1**). Furthermore, treatment decisions within each age category should also take account of factors such as ovarian reserve, previous treatment response, semen-related factors, relevant comorbidities and pregnancy risk, patient-defined reproductive goals, emotional readiness, access and cost.

Given the age-related decline in fertility and time pressure, we recommend that all women ≥35 years should receive expedited evaluation and treatment after 6–12 months of failed conception attempts, or earlier if clinically indicated. Time can be optimized by smart streamlining clinical workflows: fast-tracking appointments, particularly the first one, based on age; expediting the diagnostic process; and focusing on essential evidence-based assessments that confirm prognosis. We acknowledge that prioritizing rapid access to MAR for ARA women must be balanced with equitable access for all eligible patients within finite service capacity, and that local pathway implementation will require careful service-level planning to ensure that fast-tracking by age does not disadvantage other patient groups.

Thorough assessment for male infertility is also an essential, although frequently overlooked, part of the evaluation. Age-related decline in male fertility can contribute to a longer time to conception, higher miscarriage rates, reduced sperm quality, and increased genetic risks (35). In addition to conventional analysis of sperm parameters, such as sperm count, morphology, motility and volume, the assessment should also include paternal age, endocrine function, quality of sperm DNA, lifestyle influences and environmental determinants (36–42).

For women in the 40–43 age bracket, this timeline needs to be accelerated, particularly if >1 child is the ultimate goal. Options for oocyte freezing and/or banking should be offered and discussed as a potential way to safeguard oocyte quality.

In light of the unfavorable benefit–risk profile from the chance of success balanced against the maternal health risks (8), women > 44 years should be counselled to consider oocyte donor cycles to achieve their desired family size (43).

### Psychological support

Across all age groups, appropriate evidence-based counselling regarding age-related fertility decline and the importance of timely, informed intervention, the benefits and risks of oocyte banking, the likelihood of success associated with these methods, and the financial and ethical implications should be integrated into the treatment pathway. These discussions should also address the emotional challenges associated with infertility and MAR in ARA women (44), as well as the potential for age-related comorbidities and increased awareness of the risk factors that may affect fertility or pregnancy outcomes.

### Treatment strategy

#### Reduce time to pregnancy

Time to pregnancy is inherently age dependent and should be prioritized according to desired family size and patient age. For example, a woman aged between 35 and 39 years who desires only one child to complete their family should still receive expedited access to assessment and treatment, but there would be a greater urgency to access the treatment pathway for a woman of the same age who desires >1 child or a woman who is older (40–43 years) who desires either 1 or >1 child.

#### Maximize the pregnancy potential of each MAR cycle

Real-world data show a correlation between the number of oocytes retrieved in a stimulated cycle and improved outcomes (45), with a strong association between the number of oocytes and live births per fresh cycle (29, 46–54). Reported cumulative live birth rate data, which is defined as the first live birth following the use of all fresh and frozen embryos obtained from a single OS cycle, confirms that a higher oocyte count is associated with better overall success, potentially minimizing the need for further OS cycles (49, 51, 53, 55–58). However, to increase the probability of completing their family in the available timescale, the target number of oocytes should be based on the potential of each woman and aim to obtain as many oocytes as possible so as to give an ARA women the best chance of achieving their family goals, which may be lower than the recommended optimum of 15 oocytes in ARA women (29), and may require more than one OS cycle. Notwithstanding the need to maximize each MAR cycle, we acknowledge that high oocyte numbers do not automatically translate into increased live births for ARA women and the relations between oocyte number and euploid blastocysts, miscarriages, treatments, costs and dropouts need to be considered.

As part of the 3F framework, we have estimated the number of oocytes required to obtain a pre-specified number of euploid blastocysts (e.g., ≥1 or ≥3) for each of the proposed age groups (**Table 1**). These estimates should be interpreted advisedly, as they are based on population-level counselling strata derived from published prediction models using retrospective cohort data (31–33) and on recognized changes in reproductive potential and pregnancy-related risk. We also acknowledge that oocyte yield and modelled euploid-blastocyst yield are intermediate outcomes and are no guarantee of live birth or completion of the patient’s family-building goal.

As an example based on these modelling estimates, women aged 35–39 and 40–44 years who desire >1 child may need to bank oocytes through sequential cycles to accumulate sufficient euploid blastocysts to achieve this goal over the shortest possible timeframe (**Table 1**). Women in these age groups who only want one child to complete their family should also receive prioritized access to assessment and treatment, to mitigate any further decline in oocyte quality. Although the efficacy of Duostim remains unproven in ARA women, it may be considered to accelerate the oocyte retrieval process and potentially reduce the number of full cycles required in cases of very low ovarian reserve or an urgent need to bank embryos, if an acceptable number of euploid embryos are obtained in the initial stimulation (59).

Pre-implantation genetic testing for aneuploidy (PGT-A) is not routinely recommended as part of the 3F framework unless specifically indicated (e.g., high rates of miscarriage or recurring unsuccessful MAR cycles) (**Table 1**). This is due to the absence of evidence-based clinical data to support its effectiveness in routine clinical practice (60, 61) and the potential for otherwise viable embryos displaying mosaicism to be deprioritized and transferred when only no fully euploid embryos are available (62–66). Furthermore, the substantial additional financial burden incurred by PGT-A (67) may present a substantial opportunity cost for ARA women, reducing the total number of cycles that can be achieved (68). Overall, any decision to go ahead with PGT-A should be individualized through shared decision-making, taking account of age, ovarian reserve, embryo availability, previous reproductive history, potential benefits and limitations, cost and patient preferences.

While the intention to pursue more than one cycle may seem counterintuitive, timing is one factor that all clinicians can control. By banking oocytes or viable embryos, when appropriate, and accepting from the outset that more than one round of OS will be needed, adopting the 3F approach may still enable a high number of good-quality oocytes to be obtained safely in ARA women (53, 58), which, in turn, may increase their chances of having the desired number of children. Furthermore, through careful embryo selection and single embryo transfer, the 3F approach should also aim to minimize the risks associated with multiple pregnancies.

#### Optimize gonadotropin regimens

OS regimens should aim to optimize oocyte yield and quality, while always following best practices; however, achieving the optimal or maximum number of oocytes/euploid blastocysts presents unique challenges for ARA women. Various strategies have been suggested for personalizing gonadotropin regimens (**Table 1**). Ultimately, gonadotrophin choice should be guided by availability, convenience, cost and predictability of response. Accordingly, the weight we place on the below treatment recommendations reflect the opinion and clinical experience of the authors, corroborated by relevant published evidence, rather than a comparison of all available formulations and, as is the case with every opinion article, should be considered in the context of the funding source disclosed.

#### Type of gonadotropin

When selecting gonadotropins for use in ARA women, several key attributes must be considered to optimize OS and treatment outcomes. Potency and biological activity determine the efficacy, effectiveness and dosing of gonadotropins for OS. For example, recombinant human FSH (r-hFSH) has a high potency due to its purity, glycosylation composition and standardized formulation, and prospective randomized trials on its use in OS suggest a higher number of oocytes at lower r-hFSH doses compared with non-recombinant FSH preparations (69–74). However, a recent Cochrane Systematic Review reports that clinical pregnancy and live birth after fresh transfer are probably modestly lower with r-hFSH compared with HMG/HP-HMG, although there is probably little or no difference in cumulative ongoing pregnancy/live birth between the two preparations (75).

Quality attributes such as consistency and purity also play a significant role in effectiveness, with recombinant gonadotropins offering superior consistency and reduced variability compared with urinary-derived options, enhancing the predictability in treatment responses (76). However, all r-hFSH products may not be considered equally effective. Recently published evidence from a meta-analysis, which was independently conducted, PROSPERO-registered, unfunded and graded as of moderate-quality evidence, has reported differences in live birth rates for biosimilars of the originator r-hFSH compared with the originator product (77–80). A similar higher live birth rate with r-hFSH compared with biosimilars has also been reported in a recent Cochrane systematic review, which included the findings of Kiose et al. (75).

Finally, there is emerging evidence from a retrospective cohort database analysis (81) and a systematic review and meta-analysis (82) that co-treatment with r-hFSH and r-hLH may be beneficial for ARA women by supporting follicular development at the granulosa cell level, improving oocyte quality and reproductive outcomes. Although r-hFSH and recombinant human luteinizing hormone (r-hLH) co-treatment has been included as a conditional recommendation in the latest ESHRE Guidelines on OS (34), additional studies are needed to confirm whether this translates into increased live birth rates.

#### Starting dose

Optimizing the starting dose of gonadotropins is crucial, particularly for low responders, which includes most ARA women (83). Evidence from randomized controlled trials, summarized in recent Cochrane reviews, shows that higher doses—such as 225–300 IU per day—can lead to a greater number of oocytes retrieved compared with the standard 150 IU per day dose (84, 85). However, to avoid cycle cancellation due to ovarian hyperstimulation syndrome, the choice of starting dose should be considered according to an individual’s predicted response. Furthermore, an increase in oocyte yield may or may not correlate with improved cumulative outcomes, and further research is needed to support the use of tailored dosing strategies for this population (84–86).

#### Within-cycle dose adjustments

Modelling studies, biomarker analyses, literature reviews and retrospective analyses suggest that gonadotropin dose can be adjusted during OS based on response (84, 87–92). A retrospective analysis reported that within-cycle dose adjustment were reported to occur in approximately 40–45% of OS cycles (91, 92). However, the effects of within-cycle dose adjustments on live-birth outcomes still need further investigation. As this strategy is probably not recommended according to current guidelines, the starting dose should be rigorously selected based on patient characteristics and desired outcome (34).

#### Excess embryo policy

While the 3F protocol is designed to provide sufficient embryos to meet the desired family size for each ARA woman, there remains the potential for the creation of supernumerary embryos in the process of meeting this goal. We recommend that dispositional discussions are included as part of the pre-treatment counselling process, according to local regulations and legislation, to determine the parent’s attitudes and beliefs regarding discarding the embryos (if permitted) or retaining them in case individual family expectations change (93). Furthermore, as these embryos were obtained from ARA women, there will be practical and ethical issues surrounding the donation of supernumerary embryos to childless couples or for research purposes, if permitted.

## Conclusions and future directions

The hypothesis-generating 3F approach concept presented here recognizes the importance of tailored and realistic approaches in fertility care for the growing sub-population of ARA women seen at our clinics. In particular, it considers the specific challenges faced by ARA women, such as declining ovarian reserve and increased aneuploidy rates, while minimizing the overall impact of fertility treatments, as an achievable and time-efficient strategy for MAR in this population.

Decreased oocyte quantity and quality are hallmark features of ovarian aging and have a significant negative impact on clinical outcomes in ARA women (94). This highlights the urgent need for research focused on understanding the underlying mechanisms of ovarian aging and, particularly, how changes in oocyte-intrinsic pathways and the ovarian environment both drive ovarian aging (95). Developing novel models and identifying future therapeutic strategies that extend reproductive longevity and overall health is an urgent priority in this field.

The validity of the 3F approach in clinical practice will now need to be assessed, in the first instance, in prospective studies that assess patient-relevant outcomes, including cumulative live birth and time to live birth, miscarriage, number of stimulation cycles and procedures, adverse events, emotional burden and treatment discontinuation. Cost-effectiveness studies will also need to be conducted to ensure that any potential financial implications do not impede its wider implementation.

## Data Availability

The original contributions presented in the study are included in the article/**Supplementary Material**. Further inquiries can be directed to the corresponding author.

## Supplementary material

Supplementary Figure 1 Strategies for reducing the time to live birth in ARA women.

Supplementary Figure 2 Hypothetical case study of an ARA woman’s journey through the 3F approach.

## References

[1] Statista . Estimated average age of women at the birth of their first child in the Asia-Pacific region between 2015 and 2020, by country (2022). Available online at: https://www.statista.com/statistics/1345930/apac-mean-age-of-women-first-childbirth-by-country/ (Accessed December 5, 2024).

[2] Eurostat . Fertility Statistics (2025). Available online at: https://ec.europa.eu/eurostat/statistics-explained/index.php?title=Fertility_statistics (Accessed December 5, 2024).

[3] Eurostat . Women in the EU are Having Their First Child Later (2021). Available online at: https://ec.europa.eu/eurostat/web/products-eurostat-news/-/ddn-20210224-1 (Accessed January 27, 2025).

[4] ACOG . Pregnancy at age 35 years or older: ACOG Obstetric Care Consensus No. 11. Obstet Gynecol. (2022) 140:348–66. doi: 10.1097/AOG.0000000000004873 35852294

[5] Zabak S Varma A Bansod S Pohane MR . Exploring the complex landscape of delayed childbearing: factors, history, and long-term implications. Cureus. (2023) 15:e46291. doi: 10.7759/cureus.46291 37915872 PMC10616531

[6] McMahon CA Boivin J Gibson FL Hammarberg K Wynter K Saunders D . Age at first birth, mode of conception and psychological wellbeing in pregnancy: findings from the parental age and transition to parenthood Australia (PATPA) study. Hum Reprod. (2011) 26:1389–98. doi: 10.1093/humrep/der076 21441544

[7] Sunkara SK Zheng W D'Hooghe T Longobardi S Boivin J . Time as an outcome measure in fertility-related clinical studies: long-awaited. Hum Reprod. (2020) 35:1732–9. doi: 10.1093/humrep/deaa138 32644107 PMC7398622

[8] Ye X Baker PN Tong C . The updated understanding of advanced maternal age. Fundam Res. (2024) 4:1719–28. doi: 10.1016/j.fmre.2023.09.013 39734537 PMC11670706

[9] Franasiak JM Forman EJ Hong KH Werner MD Upham KM Treff NR . The nature of aneuploidy with increasing age of the female partner: a review of 15,169 consecutive trophectoderm biopsies evaluated with comprehensive chromosomal screening. Fertil Steril. (2014) 101:656–63. doi: 10.1016/j.fertnstert.2013.11.004 24355045

[10] Jaswa EG McCulloch CE Simbulan R Cedars MI Rosen MP . Diminished ovarian reserve is associated with reduced euploid rates via preimplantation genetic testing for aneuploidy independently from age: evidence for concomitant reduction in oocyte quality with quantity. Fertility Sterility. (2021) 115:966–73. doi: 10.1016/j.fertnstert.2020.10.051 33583594

[11] Sauer MV . Reproduction at an advanced maternal age and maternal health. Fertil Steril. (2015) 103:1136–43. doi: 10.1016/j.fertnstert.2015.03.004 25934599

[12] Johnson JA Tough S . Delayed child-bearing. J Obstet Gynaecol Can. (2012) 34:80–93. doi: 10.1016/s1701-2163(16)35138-6 22260768

[13] CDC . Centers for Disease Control ART Data (2020). Available online at: http://nccd.cdc.gov/drh_art (Accessed January 27, 2025).

[14] Smeenk J Wyns C De Geyter C Kupka M Bergh C Cuevas Saiz I . ART in Europe 2019: results generated from European registries by ESHRE†. Hum Reprod. (2023) 38:2321–38. doi: 10.1093/humrep/dead197 37847771 PMC10694409

[15] Tan TY Lau SK Loh SF Tan HH . Female ageing and reproductive outcome in assisted reproduction cycles. Singapore Med J. (2014) 55:305–9. doi: 10.11622/smedj.2014081 25017405 PMC4294057

[16] Lazzari E Potančoková M Sobotka T Gray E Chambers GM . Projecting the contribution of assisted reproductive technology to completed cohort fertility. Popul Res Policy Rev. (2023) 42:6. doi: 10.1007/s11113-023-09765-3 36789330 PMC9912242

[17] Choi SKY Lazzari E Venetis C Chambers GM . Childbirth timing and completed family size by the mode of conception-the role of medically assisted reproduction: a population-based cohort study in Australia. Lancet Reg Health West Pac. (2023) 33:100686. doi: 10.1016/j.lanwpc.2023.100686 37181531 PMC10166997

[18] De Neubourg D Bogaerts K Anagnostou E Autin C Blockeel C Coetsier T . Evolution of cumulative live birth and dropout rates over six complete IVF/ICSI cycles: a large prospective cohort study. Reprod BioMed Online. (2021) 42:717–24. doi: 10.1016/j.rbmo.2021.01.005 33518469

[19] Gossett DR Nayak S Bhatt S Bailey SC . What do healthy women know about the consequences of delayed childbearing? J Health Commun. (2013) 18 Suppl 1:118–28. doi: 10.1080/10810730.2013.825677 24093350 PMC3814907

[20] Deatsman S Vasilopoulos T Rhoton-Vlasak A . Age and fertility: a study on patient awareness. JBRA Assist Reprod. (2016) 20:99–106. doi: 10.5935/1518-0557.20160024 27584600 PMC5264372

[21] Meissner C Schippert C von Versen-Höynck F . Awareness, knowledge, and perceptions of infertility, fertility assessment, and assisted reproductive technologies in the era of oocyte freezing among female and male university students. J Assist Reprod Genet. (2016) 33:719–29. doi: 10.1007/s10815-016-0717-1 27125212 PMC4889488

[22] García D Brazal S Rodríguez A Prat A Vassena R . Knowledge of age-related fertility decline in women: a systematic review. Eur J Obstet Gynecol Reprod Biol. (2018) 230:109–18. doi: 10.1016/j.ejogrb.2018.09.030 30248536

[23] Trawick E Pecoriello J Quinn G Goldman KN . Guidelines informing counseling on female age-related fertility decline: a systematic review. J Assist Reprod Genet. (2021) 38:41–53. doi: 10.1007/s10815-020-01967-4 33188440 PMC7822973

[24] Anderson RA Amant F Braat D D'Angelo A Chuva de Sousa Lopes SM Demeestere I . ESHRE guideline: female fertility preservation†. Hum Reprod Open. (2020) 2020. doi: 10.1093/hropen/hoaa052 33225079 PMC7666361

[25] ASRM . Assisted reproduction with advancing paternal and maternal age: an Ethics Committee opinion. Fertility Sterility. (2025) 123:999–1005. doi: 10.1016/j.fertnstert.2025.03.031 40323247

[26] Huttler A Duvall D Sakkas D Heyward Q Sabbagh R Alper M . Achieving two live births from one ovarian stimulation cycle: the one-and-done approach revisited. Fertil Steril. (2026) 125:73–82. doi: 10.1016/j.fertnstert.2025.07.029 40712966

[27] Vermey BG Chua SJ Zafarmand MH Wang R Longobardi S Cottell E . Is there an association between oocyte number and embryo quality? A systematic review and meta-analysis. Reprod BioMed Online. (2019) 39:751–63. doi: 10.1016/j.rbmo.2019.06.013 31540848

[28] Venetis CA Tilia L Panlilio E Kan A . Is more better? A higher oocyte yield is independently associated with more day-3 euploid embryos after ICSI. Hum Reprod. (2019) 34:79–83. doi: 10.1093/humrep/dey342 30476100

[29] Sunkara SK Rittenberg V Raine-Fenning N Bhattacharya S Zamora J Coomarasamy A . Association between the number of eggs and live birth in IVF treatment: an analysis of 400 135 treatment cycles. Hum Reprod. (2011) 26:1768–74. doi: 10.1093/humrep/der106 21558332

[30] Paul RC Fitzgerald O Lieberman D Venetis C Chambers GM . Cumulative live birth rates for women returning to ART treatment for a second ART-conceived child. Hum Reprod. (2020) 35:1432–40. doi: 10.1093/humrep/deaa030 32380547

[31] Esteves SC Carvalho JF Bento FC Santos J . A novel predictive model to estimate the number of mature oocytes required for obtaining at least one euploid blastocyst for transfer in couples undergoing *in vitro* fertilization/intracytoplasmic sperm injection: the ART Calculator. Front Endocrinol (Lausanne). (2019) 10:99. doi: 10.3389/fendo.2019.00099 30873117 PMC6403136

[32] Esteves SC Yarali H Ubaldi FM Carvalho JF Bento FC Vaiarelli A . Validation of ART Calculator for Predicting the Number of Metaphase II Oocytes Required for Obtaining at Least One Euploid Blastocyst for Transfer in Couples Undergoing *in vitro* Fertilization/Intracytoplasmic Sperm Injection. Front Endocrinol (Lausanne). (2019) 10:917. doi: 10.3389/fendo.2019.00917 32038484 PMC6992582

[33] Namath A Flannagan K Pirtea P Toner JP Devine K . The number of autologous, vitrified mature oocytes needed to obtain three euploid blastocysts increases with age. Fertil Steril. (2025) 124:487–95. doi: 10.1016/j.fertnstert.2025.04.023 40280226

[34] ESHRE . Guideline on Ovarian Stimualation for IVF/ICSI (2025). Available online at: https://www.eshre.eu/Guidelines-and-Legal/Guidelines/Ovarian-Stimulation-in-IVF-ICSI (Accessed December 3, 2025).

[35] Harris ID Fronczak C Roth L Meacham RB . Fertility and the aging male. Rev Urol. (2011) 13:e184–90. PMC3253726 22232567

[36] Morris ID Ilott S Dixon L Brison DR . The spectrum of DNA damage in human sperm assessed by single cell gel electrophoresis (Comet assay) and its relationship to fertilization and embryo development. Hum Reprod. (2002) 17:990–8. doi: 10.1093/humrep/17.4.990 11925396

[37] Frattarelli JL Miller KA Miller BT Elkind-Hirsch K Scott RT . Male age negatively impacts embryo development and reproductive outcome in donor oocyte assisted reproductive technology cycles. Fertil Steril. (2008) 90:97–103. doi: 10.1016/j.fertnstert.2007.06.009 17765235

[38] Robertshaw I Khoury J Abdallah ME Warikoo P Hofmann GE . The effect of paternal age on outcome in assisted reproductive technology using the ovum donation model. Reprod Sci. (2014) 21:590–3. doi: 10.1177/1933719113506497 24142846

[39] García-Ferreyra J Luna D Villegas L Romero R Zavala P Hilario R . High aneuploidy rates observed in embryos derived from donated oocytes are related to male aging and high percentages of sperm DNA fragmentation. Clin Med Insights Reprod Health. (2015) 9:21–7. doi: 10.4137/cmrh.s32769 26604851 PMC4642825

[40] WHO . WHO laboratory manual for the examination and processing of human semen, 6th ed. Geneva, Switzerland: World Health Organization (2021).

[41] Esteves SC Humaidan P Ubaldi FM Alviggi C Antonio L Barratt CLR . APHRODITE criteria: addressing male patients with hypogonadism and/or infertility owing to altered idiopathic testicular function. Reprod BioMed Online. (2024) 48:103647. doi: 10.1016/j.rbmo.2023.103647 38367592

[42] Bier S Wolff A Zitzmann M Kliesch S . Normozoospermic men in infertile couples: Potential benefit of early medical diagnostic procedures. Andrology. (2025) 14(1):101–8. doi: 10.1111/andr.70035 40105106 PMC12670473

[43] Bruckamp L Lazzari E . Shifting the reproductive window: The contribution of ART and egg donation to fertility rates in the UK. Popul Stud (Camb). (2025) 80(2):321–33. doi: 10.1080/00324728.2025.2561595 41140284 PMC7618353

[44] Gameiro S Boivin J Dancet E de Klerk C Emery M Lewis-Jones C . ESHRE guideline: routine psychosocial care in infertility and medically assisted reproduction-a guide for fertility staff. Hum Reprod. (2015) 30:2476–85. doi: 10.1093/humrep/dev177 26345684

[45] Sunkara SK . Number of oocytes and IVF outcomes: real-world evidence. Best Pract Res Clin Obstet Gynaecol. (2023) 89:102341. doi: 10.1016/j.bpobgyn.2023.102341 37336119

[46] Briggs R Kovacs G MacLachlan V Motteram C Baker HWG . Can you ever collect too many oocytes? Hum Reprod. (2014) 30:81–7. doi: 10.1093/humrep/deu272 25362088

[47] Steward RG Lan L Shah AA Yeh JS Price TM Goldfarb JM . Oocyte number as a predictor for ovarian hyperstimulation syndrome and live birth: an analysis of 256,381 *in vitro* fertilization cycles. Fertil Steril. (2014) 101:967–73. doi: 10.1016/j.fertnstert.2013.12.026 24462057

[48] Nelson SM Smith A Tilling K Lawlor DA . Optimal oocyte yield for cumulative live-birth rate: analysis of 257,398 IVF cycles and their linked fresh and frozen embryo transfers. Fertility Sterility. (2016) 106:e191–2. doi: 10.1016/j.fertnstert.2016.07.55938826717

[49] Chen YH Wang Q Zhang YN Han X Li DH Zhang CL . Cumulative live birth and surplus embryo incidence after frozen-thaw cycles in PCOS: how many oocytes do we need? J Assist Reprod Genet. (2017) 34:1153–9. doi: 10.1007/s10815-017-0959-6 28580513 PMC5581780

[50] López-Rioja MJ Campos-Cañas JA Recio-López Y Quiroz-Garza G Sánchez-González CM Hinojosa-Rodríguez K . Optimal number of oocytes: *in vitro* fertilization predictive model. Ginecol Obstet Mex. (2017) 85:735–47.

[51] Magnusson Å Källen K Thurin-Kjellberg A Bergh C . The number of oocytes retrieved during IVF: a balance between efficacy and safety. Hum Reprod. (2018) 33:58–64. doi: 10.1093/humrep/dex334 29136154

[52] Polyzos NP Drakopoulos P Parra J Pellicer A Santos-Ribeiro S Tournaye H . Cumulative live birth rates according to the number of oocytes retrieved after the first ovarian stimulation for *in vitro* fertilization/intracytoplasmic sperm injection: a multicenter multinational analysis including ∼15,000 women. Fertil Steril. (2018) 110:661–670.e661. doi: 10.1016/j.fertnstert.2018.04.039 30196963

[53] Law YJ Zhang N Kolibianakis EM Costello MF Keller E Chambers GM . Is there an optimal number of oocytes retrieved at which live birth rates or cumulative live birth rates per aspiration are maximized after ART? A systematic review. Reprod BioMed Online. (2021) 42:83–104. doi: 10.1016/j.rbmo.2020.10.008 33390313

[54] Zhang N Law YJ Venetis CA Chambers GM Harris K . Female age is associated with the optimal number of oocytes to maximize fresh live birth rates: an analysis of 256,643 fresh ART cycles. Reprod BioMed Online. (2021) 42:669–78. doi: 10.1016/j.rbmo.2020.11.010 33509664

[55] Ji J Liu Y Tong XH Luo L Ma J Chen Z . The optimum number of oocytes in IVF treatment: an analysis of 2455 cycles in China. Hum Reprod. (2013) 28:2728–34. doi: 10.1093/humrep/det303 23887075

[56] Drakopoulos P Blockeel C Stoop D Camus M de Vos M Tournaye H . Conventional ovarian stimulation and single embryo transfer for IVF/ICSI. How many oocytes do we need to maximize cumulative live birth rates after utilization of all fresh and frozen embryos? Hum Reprod. (2016) 31:370–6. doi: 10.1093/humrep/dev316 26724797

[57] Devesa M Tur R Rodríguez I Coroleu B Martínez F Polyzos NP . Cumulative live birth rates and number of oocytes retrieved in women of advanced age. A single centre analysis including 4500 women ≥38 years old. Hum Reprod. (2018) 33:2010–7. doi: 10.1093/humrep/dey295 30272168

[58] Law YJ Zhang N Venetis CA Chambers GM Harris K . The number of oocytes associated with maximum cumulative live birth rates per aspiration depends on female age: a population study of 221 221 treatment cycles. Hum Reprod. (2019) 34:1778–87. doi: 10.1093/humrep/dez100 31398253

[59] Vaiarelli A Cimadomo D Petriglia C Conforti A Alviggi C Ubaldi N . DuoStim - a reproducible strategy to obtain more oocytes and competent embryos in a short time-frame aimed at fertility preservation and IVF purposes. A systematic review. Ups J Med Sci. (2020) 125:121–30. doi: 10.1080/03009734.2020.1734694 32338123 PMC7721001

[60] Lensen S Wilkinson J Steeper M Melo P Kamath MS Craciunas L . Safety and effectiveness of ten common in-vitro fertilisation add-ons: a systematic review and meta-analysis. Lancet Obstetrics Gynaecology Women’s Health. (2026) 2:e624–36. doi: 10.1016/s3050-5038(26)00054-3

[61] Roberts SA Brison DR Harper J Kondowe F Vail A . PGT-A is associated with a reduced live birth rate in routine practice: evidence from the UK ART register. Reprod BioMed Online. (2026) 53:105747. doi: 10.1016/j.rbmo.2026.105747 42275751

[62] Leigh D Cram DS Rechitsky S Handyside A Wells D Munne S . PGDIS position statement on the transfer of mosaic embryos 2021. Reprod BioMed Online. (2022) 45:19–25. doi: 10.1016/j.rbmo.2022.03.013 35523707

[63] ASRM . Clinical management of mosaic results from preimplantation genetic testing for aneuploidy of blastocysts: a committee opinion. Fertility Sterility. (2023) 120:973–82. doi: 10.1016/j.fertnstert.2023.08.969 37678731

[64] Lundin K Bentzen JG Bozdag G Ebner T Harper J Le Clef N . Good practice recommendations on add-ons in reproductive medicine†. Hum Reprod. (2023) 38:2062–104. doi: 10.1093/humrep/dead184 37747409 PMC10628516

[65] ASRM . The use of preimplantation genetic testing for aneuploidy: a committee opinion. Fertil Steril. (2024) 122:421–34. doi: 10.1016/j.fertnstert.2024.04.013 38762806

[66] HFEA . Treatment Add-Ons with Limited Evidence (2025). Available online at: https://www.hfea.gov.uk/treatments/treatment-add-ons/#treatment-add-ons (Accessed December 12, 2025).

[67] Goldman RH Racowsky C Farland LV Fox JH Munné S Ribustello L . The cost of a euploid embryo identified from preimplantation genetic testing for aneuploidy (PGT-A): a counseling tool. J Assist Reprod Genet. (2018) 35:1641–50. doi: 10.1007/s10815-018-1275-5 30066304 PMC6133815

[68] Viville S Aboulghar M . PGT-A: what's it for, what's wrong? J Assist Reprod Genet. (2025) 42:63–9. doi: 10.1007/s10815-025-03400-0 39847200 PMC11806166

[69] Andersen AN Devroey P Arce JC . Clinical outcome following stimulation with highly purified hMG or recombinant FSH in patients undergoing IVF: a randomized assessor-blind controlled trial. Hum Reprod. (2006) 21:3217–27. doi: 10.1093/humrep/del284 16873892

[70] Bosch E Vidal C Labarta E Simon C Remohi J Pellicer A . Highly purified hMG versus recombinant FSH in ovarian hyperstimulation with GnRH antagonists--a randomized study. Hum Reprod. (2008) 23:2346–51. doi: 10.1093/humrep/den220 18583332

[71] Hompes PG Broekmans FJ Hoozemans DA Schats R . Effectiveness of highly purified human menopausal gonadotropin vs. recombinant follicle-stimulating hormone in first-cycle *in vitro* fertilization-intracytoplasmic sperm injection patients. Fertil Steril. (2008) 89:1685–93. doi: 10.1016/j.fertnstert.2007.05.039 17681325

[72] Devroey P Pellicer A Nyboe Andersen A Arce JC . A randomized assessor-blind trial comparing highly purified hMG and recombinant FSH in a GnRH antagonist cycle with compulsory single-blastocyst transfer. Fertil Steril. (2012) 97:561–71. doi: 10.1016/j.fertnstert.2011.12.016 22244781

[73] Ye H Huang G Pei L Zeng P Luo X . Outcome of *in vitro* fertilization following stimulation with highly purified hMG or recombinant FSH in downregulated women of advanced reproductive age: a prospective, randomized and controlled trial. Gynecol Endocrinol. (2012) 28:540–4. doi: 10.3109/09513590.2011.650742 22390186

[74] Witz CA Daftary GS Doody KJ Park JK Seifu Y Yankov VI . Randomized, assessor-blinded trial comparing highly purified human menotropin and recombinant follicle-stimulating hormone in high responders undergoing intracytoplasmic sperm injection. Fertil Steril. (2020) 114:321–30. doi: 10.1016/j.fertnstert.2020.03.029 32416978

[75] Berkhout RP Kostova EB van Wely M . Recombinant follicle-stimulating hormone (rFSH) versus other recombinant or urinary gonadotropins for ovarian stimulation in assisted reproductive technology cycles. Cochrane Database Syst Rev. (2026) 7:CD005354. doi: 10.1002/14651858.cd005354.pub3 42445958 PMC13366490

[76] Lispi M Humaidan P Bousfield GR D'Hooghe T Ulloa-Aguirre A . Follicle-stimulating hormone biological products: does potency predict clinical efficacy? Int J Mol Sci. (2023) 24. doi: 10.3390/ijms24109020 37240364 PMC10218858

[77] de Mora F Howles CM Trew G . Follitropin alfa biosimilars: unfounded doubts on the road to reproductive care. Hum Reprod. (2025) 40:1789–90. doi: 10.1093/humrep/deaf143 40713789 PMC12408895

[78] Kiose KI Storr A Kolibianakis EM Mol BW Venetis CA . Biosimilars versus the originator of follitropin alfa for ovarian stimulation in ART: a systematic review and meta-analysis. Hum Reprod. (2025) 40:343–59. doi: 10.1093/humrep/deae274 39719046 PMC11788201

[79] Santulli P Brandão P D'Amato G Grynberg M Hamamah S Luco MI . Methodological concerns and clinical relevance: a critical appraisal of the meta-analysis on biosimilars versus originator follitropin alfa in ART. Hum Reprod. (2025) 40:1791–2. doi: 10.1093/humrep/deaf144 40713809

[80] Venetis CA Kiose KI Storr A Mol BW Kolibianakis EM . Reply: biosimilars versus the originator of follitropin alfa in ART: eyes wide shut? Hum Reprod. (2025) 40:1793–6. doi: 10.1093/humrep/deaf145 40713868

[81] Bielfeld AP Schwarze JE Verpillat P Lispi M Fischer R Hayward B . Effectiveness of recombinant human FSH: recombinant human LH combination treatment versus recombinant human FSH alone for assisted reproductive technology in women aged 35-40 years. Reprod BioMed Online. (2024) 48:103725. doi: 10.1016/j.rbmo.2023.103725 38593745

[82] Conforti A Esteves SC Humaidan P Longobardi S D'Hooghe T Orvieto R . Recombinant human luteinizing hormone co-treatment in ovarian stimulation for assisted reproductive technology in women of advanced reproductive age: a systematic review and meta-analysis of randomized controlled trials. Reprod Biol Endocrinol. (2021) 19:91. doi: 10.1186/s12958-021-00759-4 34154604 PMC8215738

[83] Ferraretti AP La Marca A Fauser BC Tarlatzis B Nargund G Gianaroli L . ESHRE consensus on the definition of 'poor response' to ovarian stimulation for *in vitro* fertilization: the Bologna criteria. Hum Reprod. (2011) 26:1616–24. doi: 10.1093/humrep/der092 21505041

[84] Lensen SF Wilkinson J Leijdekkers JA La Marca A Mol BWJ Marjoribanks J . Individualised gonadotropin dose selection using markers of ovarian reserve for women undergoing *in vitro* fertilisation plus intracytoplasmic sperm injection (IVF/ICSI). Cochrane Database Syst Rev. (2018) 2:CD012693. doi: 10.1002/14651858.CD012693.pub2 29388198 PMC6491064

[85] Ngwenya O Lensen SF Vail A Mol BWJ Broekmans FJ Wilkinson J . Individualised gonadotropin dose selection using markers of ovarian reserve for women undergoing *in vitro* fertilisation plus intracytoplasmic sperm injection (IVF/ICSI). Cochrane Database Syst Rev. (2024) 1:CD012693. doi: 10.1002/14651858.cd012693.pub3 38174816 PMC10765476

[86] Schouten N Wang R Torrance H Van Tilborg T Bastu E Bergh C . Development and validation of a gonadotropin dose selection model for optimized ovarian stimulation in IVF/ICSI: an individual participant data meta-analysis. Hum Reprod Update. (2025) 31:116–32. doi: 10.1093/humupd/dmae032 39707165 PMC11879166

[87] Nelson SM . Biomarkers of ovarian response: current and future applications. Fertil Steril. (2013) 99:963–9. doi: 10.1016/j.fertnstert.2012.11.051 23312225

[88] Sighinolfi G Grisendi V La Marca A . How to personalize ovarian stimulation in clinical practice. J Turk Ger Gynecol Assoc. (2017) 18:148–53. doi: 10.4274/jtgga.2017.0058 28890430 PMC5590212

[89] Alviggi C Conforti A Esteves SC Vallone R Venturella R Staiano S . Understanding ovarian hypo-response to exogenous gonadotropin in ovarian stimulation and its new proposed marker-the follicle-to-oocyte (FOI) index. Front Endocrinol (Lausanne). (2018) 9:589. doi: 10.3389/fendo.2018.00589 30386293 PMC6199413

[90] Lunenfeld B Bilger W Longobardi S Kirsten J D'Hooghe T Sunkara SK . Decision points for individualized hormonal stimulation with recombinant gonadotropins for treatment of women with infertility. Gynecol Endocrinol. (2019) 35:1027–36. doi: 10.1080/09513590.2019.1650345 31392906

[91] Fatemi H Bilger W Denis D Griesinger G La Marca A Longobardi S . Dose adjustment of follicle-stimulating hormone (FSH) during ovarian stimulation as part of medically-assisted reproduction in clinical studies: a systematic review covering 10 years, (2007-2017). Reprod Biol Endocrinol. (2021) 19:68. doi: 10.1186/s12958-021-00744-x 33975610 PMC8112039

[92] Mahony MC Hayward B Mottla GL Richter KS Beall S Ball GD . Recombinant human follicle-stimulating hormone alfa dose adjustment in US clinical practice: an observational, retrospective analysis of a real-world electronic medical records database. Front Endocrinol (Lausanne). (2021) 12:742089. doi: 10.3389/fendo.2021.742089 34956077 PMC8696034

[93] Bartolacci A Dolci C Pagliardini L Papaleo E . Too many embryos: a critical perspective on a global challenge. J Assist Reprod Genet. (2024) 41:1821–4. doi: 10.1007/s10815-024-03159-w 38839697 PMC11263306

[94] Wu J Liu Y Song Y Wang L Ai J Li K . Aging conundrum: a perspective for ovarian aging. Front Endocrinol (Lausanne). (2022) 13:952471. doi: 10.3389/fendo.2022.952471 36060963 PMC9437485

[95] Babayev E Duncan FE . Age-associated changes in cumulus cells and follicular fluid: the local oocyte microenvironment as a determinant of gamete quality. Biol Reprod. (2022) 106:351–65. doi: 10.1093/biolre/ioab241 34982142 PMC8862720

